# Clinical Characteristics and Disease Predictors of a Large Chinese Cohort of Patients with Autosomal Dominant Polycystic Kidney Disease

**DOI:** 10.1371/journal.pone.0092232

**Published:** 2014-03-20

**Authors:** Dongping Chen, Yiyi Ma, Xueqi Wang, Shengqiang Yu, Lin Li, Bing Dai, Zhiguo Mao, Lijun Sun, Chenggang Xu, Shu Rong, Mengjun Tang, Hongbo Zhao, Hongchao Liu, Andreas L. Serra, Nicole Graf, Shiyuan Liu, Rudolf P. Wüthrich, Changlin Mei

**Affiliations:** 1 Kidney Institute, Department of Nephrology, Shanghai Changzheng Hospital, Second Military Medical University, Shanghai, P. R. China; 2 Department of Radiology, Shanghai Changzheng Hospital, Second Military Medical University, Shanghai, P. R. China; 3 Division of Nephrology, University Hospital, Zürich, Switzerland; 4 Graf biostatistics, Winterthur, Switzerland; National Institutes of Health, United States of America

## Abstract

**Objective:**

Autosomal dominant polycystic kidney disease (ADPKD) is a relentlessly progressing form of chronic kidney disease for which there is no cure. The aim of this study was to characterize Chinese patients with ADPKD and to identify the factors which predict cyst growth and renal functional deterioration.

**Methods:**

To analyze disease predicting factors we performed a prospective longitudinal observational study in a cohort of 541 Chinese patients with ADPKD and an eGFR ≥30 ml/min/1.73 m^2^. Patients were followed clinically and radiologically with sequential abdominal magnetic resonance imaging (MRI). Clinical characteristics and laboratory data were related to changes in estimated glomerular filtration rate (eGFR) and total kidney volume (TKV). A linear regression model was developed to analyze the factors which determine eGFR and TKV changes.

**Results:**

The age range of this unselected cohort ranged from 4 to 77 years. Median follow-up time was 14.3±10.6 months. Although inter-individual differences in eGFR and TKV were large, there was a consistent link between these two parameters. Baseline log_10_-transformed TKV and urinary protein/creatinine ratio were identified as the major predictors for a faster eGFR decline and were associated with a higher TKV growth rate. Interestingly, a lower thrombocyte count correlated significantly with lower eGFR (r = 0.222) and higher TKV (r = 0.134).

**Conclusions:**

This large cohort of Chinese patients with ADPKD provides unique epidemiological data for comparison with other cohorts of different ethnicity. In Chinese patients we identified a lower thrombocyte count as a significant predictor of disease progression. These results are important for the design of future clinical trials to retard polycystic kidney disease progression.

## Introduction

Autosomal dominant polycystic kidney disease (ADPKD) is a distinct genetic disease which occurs with variable frequency in all parts of the world [1], [2]. Its prevalence is estimated to average 100 cases per 100’000 population, although recent studies concluded that the prevalence is closer to orphan diseases, i.e., in the range of 50 per 100’000 [3]. Mutations in two different genes (*PKD1* and *PKD2*) are known to cause ADPKD [4], [5]. Numerous different mutations in the *PKD1* gene which encodes the ciliary protein polycystin-1 are responsible for 85% of the cases, and mutations in *PKD2* encoding polycystin-2 are responsible for the remaining 15% of the cases [6]. Mutations in both genes cause significant phenotypic variability, and *PKD2* gene mutations generally lead to milder disease than *PKD1* gene mutations [6], [7].

Although the clinical description of ADPKD is well known, it remains difficult to predict the clinical course and the occurrence of complications. Therefore longitudinal cohorts have been established to document the long-term clinical course of the disease and to identify parameters which predict progression. The best characterized cohort (n = 241) with the longest follow-up is the cohort of the Consortium for Radiologic Imaging Studies of Polycystic Kidney Disease (CRISP) in the United States which has provided important information regarding the main predictors of renal disease progression [8]–[10]. Other well characterized longitudinal cohorts have been set up, including the Swiss ADPKD (n = 100) [11] and the recently established global OVERTURE (n of ca. 3000) [12] cohorts, which will continue to provide important information regarding major determinants of disease progression. Data generated from these cohorts will also be essential to define the clinically relevant parameters which are needed to evaluate the efficacy of novel treatments.

Here we describe the clinical characteristics and the factors which predict disease progression in a unique longitudinal cohort of Chinese patients (n = 541) with ADPKD. These data will be useful to design future clinical trials which endeavor to define therapies which retard disease progression in ADPKD.

## Subjects and Methods

### Patient recruitment

The study protocol and consent form were developed, reviewed and approved by the Kidney Institute of PLA. The study was approved by the Ethics Committee of the Second Military Medical University (Shanghai, China). Chinese patients with an established diagnosis of polycystic kidney disease were identified by a nationwide search in mainland China. All patients were recruited at a single nephrology center at Changzheng Hospital (Shanghai, China). Recruitment started in June 2009, and is ongoing. Informed written consent was obtained from each potentially eligible subject or his legal representative prior to enrollment.

### Inclusion and exclusion criteria

Patients had to have an established diagnosis of autosomal dominant polycystic kidney disease (ADPKD) as defined by the criteria of Pei [13]. There were no age limits, but the eGFR had to be ≥30 ml/min/1.73 m^2^ according to the CKD-EPI formula for adults or the Schwarz equation for children. Patients on dialysis treatment or having received a kidney transplant were excluded.

Subjects were ineligible to participate if they were unable to undergo breath-held magnetic resonance imaging (MRI), or had contraindications for MRI such as having a cardiac pacemaker, metallic foreign bodies or aneurysmal clips. Patients were also excluded if they had systemic diseases other than hypertension that could potentially affect renal function. Female patients who were pregnant, lactating or less than 6 months postpartum were also excluded.

### Schedule of examinations

Medical histories were taken and physical examinations were performed at the time of screening and enrollment at Changzheng Hospital in Shanghai. Enrolled subjects were scheduled for a 2-day evaluation visit, and were then seen at 6 months intervals. Blood and urine samples were collected at each visit. Abdominal MRI scans were obtained at baseline and at each follow-up visit, using a previously described protocol [11]. Total kidney volumes (TKV) and total renal cyst volumes (TCV) were measured twice by two independent observers on an interactive workstation (Advantage Windows Workstation; GE Medical Systems Europe, Buc, France).

### Statistical analyses

All medical data were anonymized and transferred to a spreadsheet. Descriptive analyses were performed for all baseline data (mean±SD, median, range). Female and male patients were compared with a two-tailed Fisher’s exact test for nominal variables and with a two-tailed Mann-Whitney U test for quantitative variables. For the correlation analyses, the Pearson product-moment correlation coefficient or Spearman’s rho was calculated.

A multiple linear regression was run with yearly eGFR change or yearly TKV growth (%) as dependent variable, and the following independent variables: age, sex, observation time, history of hypertension, intake of antihypertensive drugs, SBP, DBP, episodes of macrohematuria, baseline eGFR, protein/creatinine ratio, baseline TKV and baseline thrombocyte count. A total of 279 patients aged >18 years and ≤60 years with valid data for all variables of the model were included. For the variables SBP, DBP, episodes of macrohematuria and thrombocyte count, missing values were imputed by data from the next following visit. Overall, the missingness of data was dependent on observation time, i.e., patients with fewer visits and thus shorter observation time had more missing data. Except for observation time, Little’s test indicated that data were missing completely at random (*P* = 0.105).

For predictor selection, a stepwise forward and backward selection using the exact Akaike Information Criterion (AIC) was performed. In the model with yearly eGFR change as dependent variable, baseline eGFR was also included as additional controlling variable. Nonlinearity was evaluated with component plus residual plots, normality was checked with a Q-Q plot for studentized residuals. Homoscedasticity was evaluated with a studentized residuals versus fitted values plot and with the non-constant error variance test. The outlier test and Cook’s D plot were used to check for outliers and influential points. Several measures had to be taken to meet the assumptions of linear regression. A normal distribution of the residuals was achieved by windsorizing the dependent variable. Linearity was reached by log_10_-transforming baseline TKV and protein/creatinine ratio. For the model with yearly TKV change as dependent variable, heterogeneity of variance could be reduced considerably by introducing observation time into the model. However, as the non-constant variance score test was still of borderline significance (*P* = 0.059), a regression with robust standard errors was run.

All analyses were done using SPSS Version 20 except for the regression, which was performed in R version 2.15.2.

## Results

### General characteristics of the cohort

The Chinese ADPKD cohort represents an ongoing longitudinal study. In the present interim report, we analyzed 541 patients who were enrolled between June 2009 and December 2011. Patients were followed up every six months. The mean follow-up duration was 14.3±10.6 months (median 16 months, range 0–38 months).

The recruitment strategy of the cohort subjects was designed to allow for the inclusion of a broad age range of patients with ADPKD, including children. Table 1 shows the baseline demographic and clinical characteristics of the cohort, stratified for male and female patients. The mean age was 40 years (range 4 to 77 years). Seventy-five percent of the patients had a positive family history for ADPKD. The average disease burden was important. Two thirds of the patients were hypertensive, taking on average 1.1±1.0 antihypertensive drugs (range 0–5). Approximately half of the patients used angiotensin converting enzyme inhibitors (ACEI) or angiotensin receptor blockers (ARB) and a quarter of the patients used calcium channel blockers (CCB) to treat hypertension. Approximately three quarters of the patients had liver cysts, 37% complained of chronic pain, and 23% have had episodes of macrohematuria. Compared to male patients, female patients had a lower BMI (*P*<0.001), were less often hypertensive (*P*<0.001), had slightly more often liver cysts (*P* = 0.444) and more pain (*P* = 0.311), but less often macrohematuria (*P* = 0.035). Frequency distributions for age, antihypertensive drugs, diastolic blood pressure (DBP), systolic blood pressure (SBP), eGFR, and TKV are shown in the Figures S1A to S1F.

**Table 1:** Baseline demographics and clinical characteristics of Chinese ADPKD cohort.

Characteristics	N	Female	Male	Total
Age (years)	541	39.2±12.3	40.0±12.0	39.7±12.1
Weight (kg)	539	56.5±9.7	70.8±11.6	64.2±12.9
Height (cm)	539	160.6±8.1	173.4±6.5	167.5±9.7
BMI (kg/m^2^)	539	21.8±3.0	23.5±3.2	22.7±3.2
SBP (mm Hg)	403	129.8±17.4	132.8±16.0	131.4±16.7
DBP (mm Hg)	403	86.6±11.4	88.1±10.1	87.4±10.7
History of hypertension	531	56.6%	76.3%	67.2%
Number of antihyper-tensive drugs	522	0.9±1.1	1.2±1.0	1.1±1.0
- ACE or ARB		45.2%	61.1%	53.8%
- CCB		22.6%	26.5%	24.7%
Previous or current smokers	513	1.7%	30.6%	17.3%
Family history for ADPKD	509	74.2%	76.4%	75.4%
Presence of liver cysts	541	73.7%	70.7%	72.1%
Presence of regular pain	509	39.5%	34.8%	36.9%
History of macro-hematuria	517	18.7%	27.5%	23.4%

Baseline demographics and clinical characteristics for female (n = 251) and male (n = 290) patients with ADPKD are reported separately, and for both sexes combined (n = 541). Data show mean ± standard deviation or relative frequency (%), respectively.

### Characteristics of renal function in the cohort

Table 2 displays the baseline renal parameters (blood and urine), stratified for six different age categories. In the age range between 19 and 60, a gradual rise in the serum levels of creatinine, cystatin C, BUN, uric acid and β_2_-microglobulin was found with increasing age. Likewise, the eGFR (creatinine-based CKD-EPI formula) showed a steady decrease in these four age categories. Correlation analysis confirmed that age was negatively correlated with eGFR (see below). Of note, the yearly eGFR decline became more pronounced with increasing age, amounting to –3.5 ml/min/1.73 m^2^ per year in the age category of 51 to 60. Furthermore, the urine protein/creatinine ratio also increased with age, whereas the urine specific gravity (1.016±0.007 g/cm^3^) and the urine pH (5.69±0.50) did not change substantially in the different age groups. Table S1 in File S1 provides additional eGFR data for reference.

**Table 2:** Baseline renal parameters and eGFR data stratified by age categories.

Age category	years	≤18	19–30	31–40	41–50	51–60	>60	All
Blood								

Baseline laboratory data and eGFR for age categories ≤18 years (n = 20), 19–30 years (n = 74), 31–40 years (n = 171), 41–50 years (n = 128), 51–60 years (n = 90), and >60 years (n = 19) are reported. To reduce the number of missing values, baseline cystatin C, β_2_-microglobulin and the protein/creatinine ratio were replaced by the mean of all measured values from all visits. Data show mean ± standard deviation.

**Figure 1:**
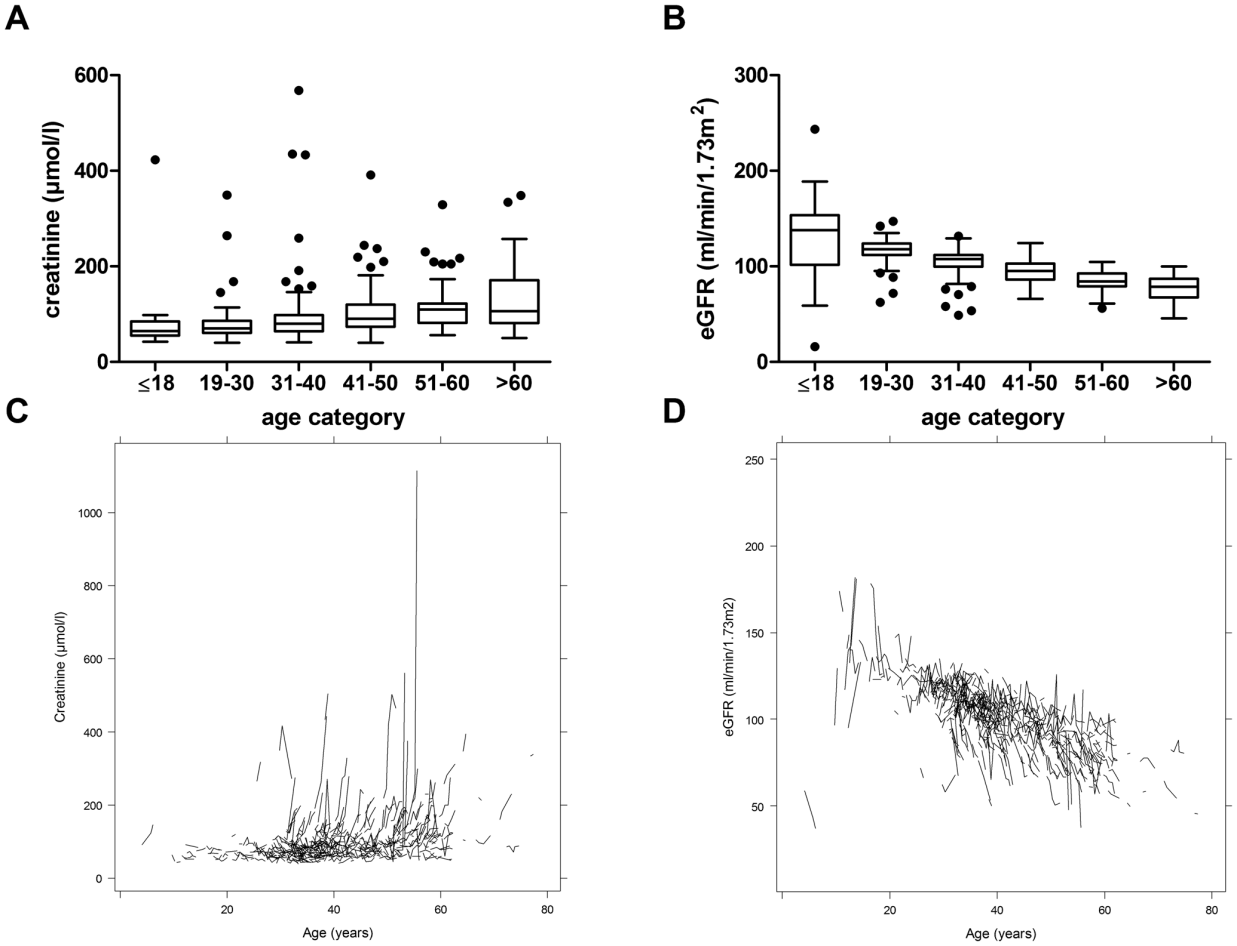
Renal function in different age categories of ADPKD patients. A) Creatinine (μmol/l) for age categories ≤18 years (n = 20), 19–30 years (n = 74), 31–40 years (n = 171), 41–50 years (n = 128), 51–60 years (n = 90), and >60 years (n = 19). B) eGFR (ml/min/1.73 m^2^) for age categories ≤18 years (n = 20), 19–30 years (n = 74), 31–40 years (n = 171), 41–50 years (n = 128), 51–60 years (n = 90), and >60 years (n = 19). Boxes show the median and the 25^th^ and 75^th^ percentile. Whiskers extend to the farthest points that are not outliers (i.e., that are within 3/2 times the interquartile range) and dots indicate outliers. C) Spaghetti plots for course of creatinine and D) eGFR over entire observation time in individual patients.

Figure 1 graphically depicts the distribution of serum creatinine and eGFR in the different age categories (Fig. 1A and 1B), and the change over time in serum creatinine and eGFR in individual patients (Fig. 1C and 1D). Overall, there was a wide spectrum of eGFR in all age categories. Of note, a pronounced creatinine rise and eGFR decline was apparent in many patients in all age categories.

**Figure 2:**
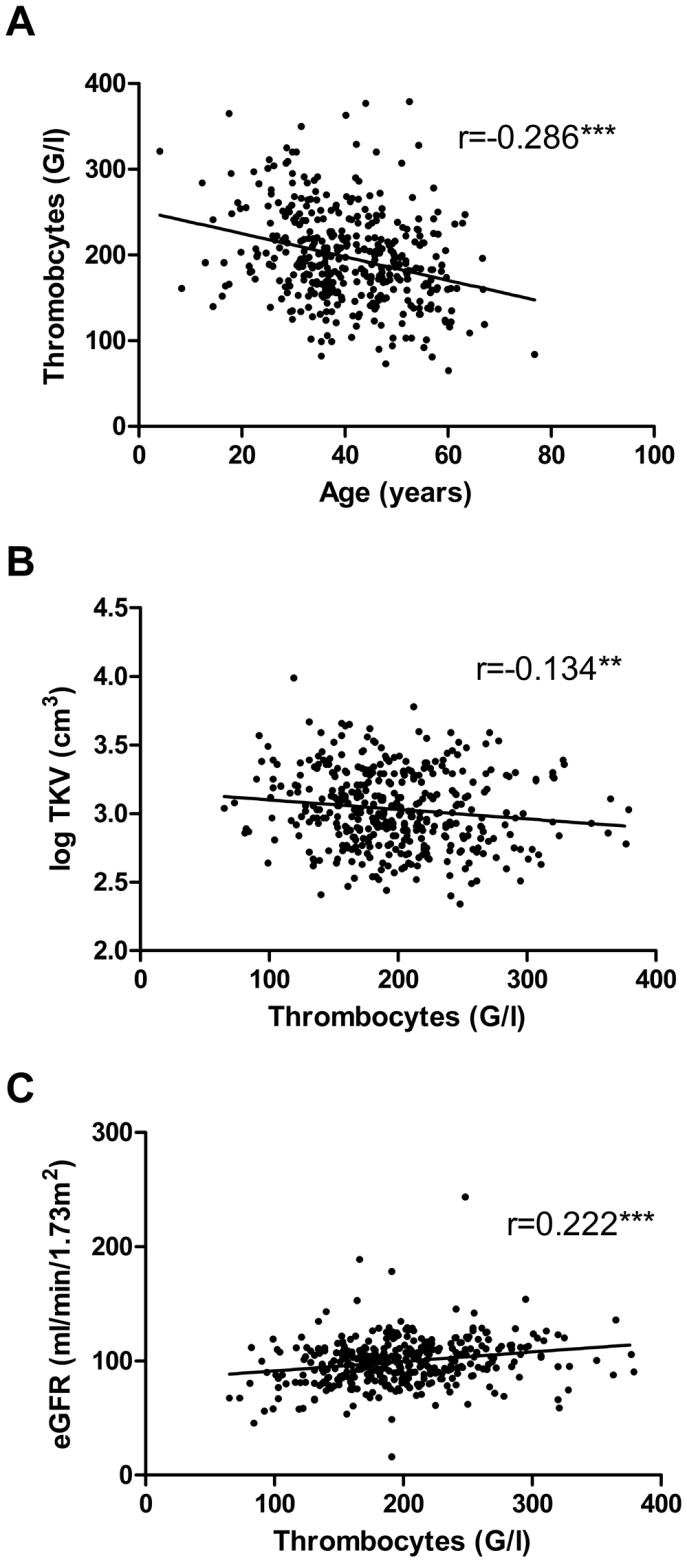
Thrombocytes were correlated to age, TKV and eGFR in ADPKD patients. A) Scatter plot between age and thrombocytes (n = 399). Pearson correlation coefficient is –0.286 (*P*<0.001). B) Scatter plot between thrombocytes and log_10_ TKV (n = 394). Pearson correlation coefficient is –0.134 (*P* = 0.008). C) Scatter plot between thrombocytes and eGFR (n = 391). Pearson correlation coefficient is 0.222 (*P*<0.001).

Regarding other laboratory data such as electrolytes, lipids and hematological parameters, no apparent differences could be detected among the different age categories, except for the hemoglobin level and more importantly the thrombocyte count which were both lower in the advanced age groups (Table S2 in File S1). The correlation analysis confirmed that the thrombocyte count was negatively correlated with age (Fig. 2A). Moreover, the thrombocyte count was found to be negatively correlated with log_10_-transformed TKV and positively with eGFR (Fig. 2B and 2C). Out of 399 patients, 60 (15.0%) had a baseline thrombocyte count below 145 G/l and were thus clearly thrombocytopenic.

### Characteristics of renal volume growth in the cohort

The mean TKV for the entire cohort amounted to 1265±1002 cm^3^ at baseline, and the mean TCV was 869±929 cm^3^, corresponding on average to 58±21% of the TKV. The mean yearly increase of TKV amounted to 72±183 cm^3^, representing 4.6±10.2% TKV growth per year (Table 3). The large standard deviations are noticeable, illustrating the large inter-individual differences of the renal volume in the cohort. On average, female patients had a lower TKV than males (1081±891 vs. 1427±1065 cm^3^, *P*<0.001), and a lower yearly TKV growth rate (3.3±11.3 vs. 5.7±9.2%, *P* = 0.003).

**Table 3:** Baseline kidney and cyst volumes and yearly volume growth rates stratified by age.

Age category	years	≤18	19–30	31–40	41–50	51–60	>60	All
**Baseline TKV**								
	N	23	81	180	134	95	19	532
	cm^3^	458±385	842±646	1126±739	1495±909	1575±948	2180±2774	1265±1002
**Yearly TKV growth**								
	N	15	61	157	99	76	13	421
	cm^3^/yr	40±174	65±153	71±185	83±193	61±154	130±328	72±183
	%/yr	2.3±24.3	4.9±10.8	5.4±9.6	4.8±9.3	3.6±7.3	1.1±11.5	4.6±10.2
**Baseline TCV**								
	N	23	81	180	134	95	19	532
	cm^3^	187±359	464±602	719±652	1072±857	1189±858	1811±2613	869±929
**Yearly TCV growth**								
	N	15	61	157	99	76	13	421
	cm^3^/yr	34±180	61±132	70±177	67±201	52±180	159±395	66±188
	%/yr	17.8±59.3	10.6±18.4	11.2±19.6	6.8±14.9	4.5±11.9	2.9±19.0	8.9±20.1

Baseline kidney volumes (TKV and TCV) and yearly volume growth rates for age categories ≤18 years, 19–30 years, 31–40 years, 41–50 years, 51–60 years, and >60 years are reported. Data show mean ± standard deviation.

**Figure 3:**
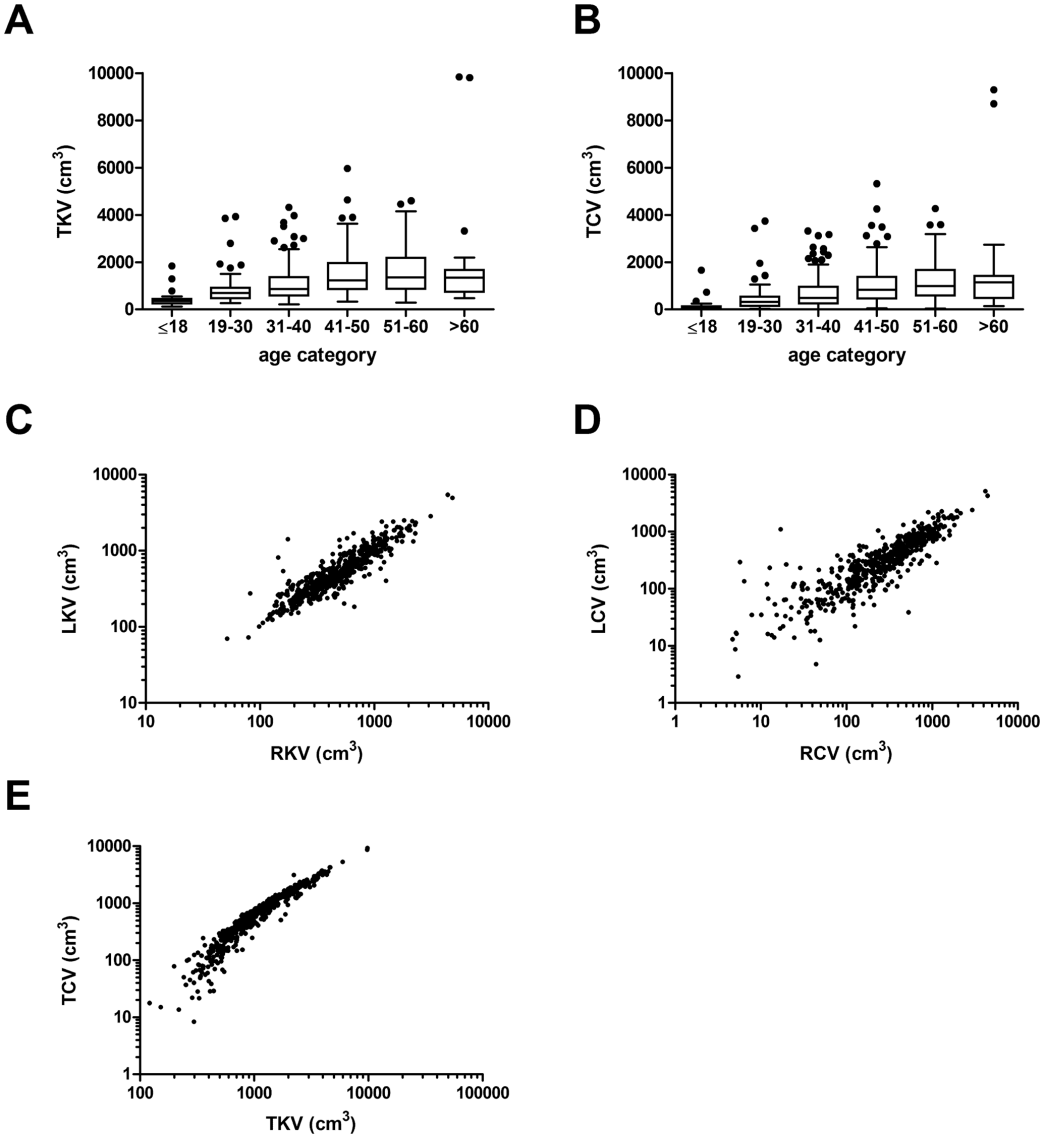
TKV and TCV in different age categories, and the correlations between kidney volumes (KV) and cyst volumes (CV). A) TKV and B) TCV for age categories ≤18 years (n = 23), 19–30 years (n = 81), 31–40 years (n = 180), 41–50 years (n = 134), 51–60 years (n = 95), and >60 years (n = 19). Boxes show the median and the 25^th^ and 75^th^ percentile. Whiskers extend to the farthest points that are not outliers (i.e., that are within 3/2 times the interquartile range) and dots indicate outliers. C) Scatter plot between RKV and LKV (n = 532). The regression line is defined by LKV = 0.337+0.889*RKV. Pearson correlation coefficient is 0.902 (*P*<0.001). D) Scatter plot between RCV and LCV (n = 530). Pearson correlation coefficient is 0.857 (*P*<0.001). E) Scatter plot between baseline TKV and baseline TCV (n = 532). Pearson correlation coefficient is 0.951 (*P*<0.001).

Figure 3A and 3B graphically depict the TKV and the TCV in the different age categories, revealing that TKV and TCV increased up to the age of 60. TKV was indeed positively correlated with age (see below). Figures 3C, 3D and 3E illustrate that there was a tight correlation between RKV and LKV (*r* = 0.902), between RCV and LCV (*r* = 0.857), and between TKV and TCV (*r* = 0.951). The Tables S3 and S4 in File S1 show additional kidney volume data for reference.

**Figure 4:**
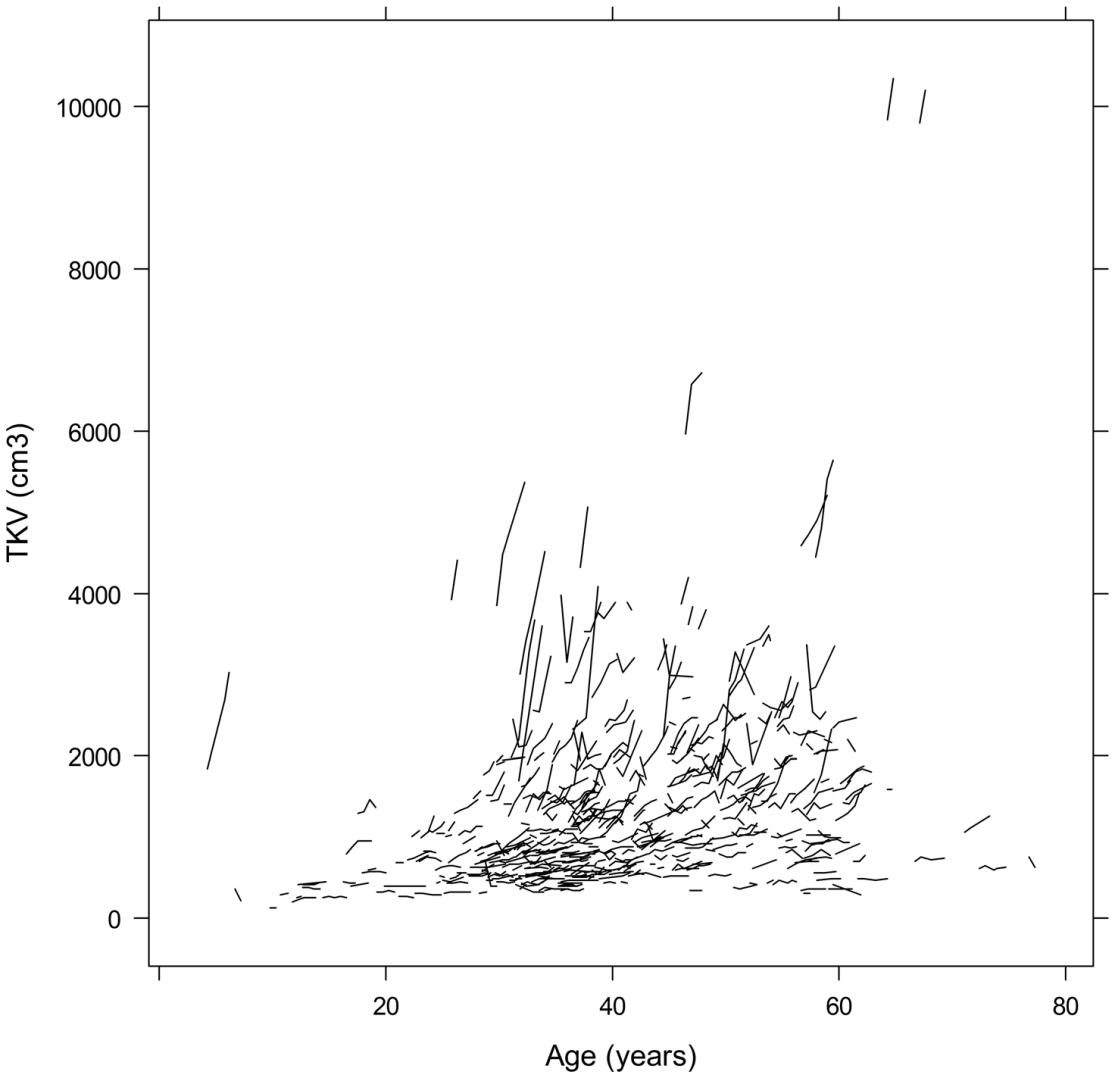
Spaghetti plot for TKV over entire observation time in individual patients (n = 421).

Figure 4 shows the TKV change over time in individual patients. Overall, the range of TKV increased with advancing age, reflecting large inter-individual differences. Of note, steep increases in TKV were found in all age categories and especially in patients with baseline TKV greater than 1500 cm^3^. In Figure S2, baseline TKV and the yearly TKV growth rate are lined up in ascending order, with the corresponding yearly TKV growth rate and baseline TKV being displayed in the graph above, illustrating that the growth rate appeared to increase with increasing baseline TKV.

### Correlations between TKV and eGFR

**Figure 5:**
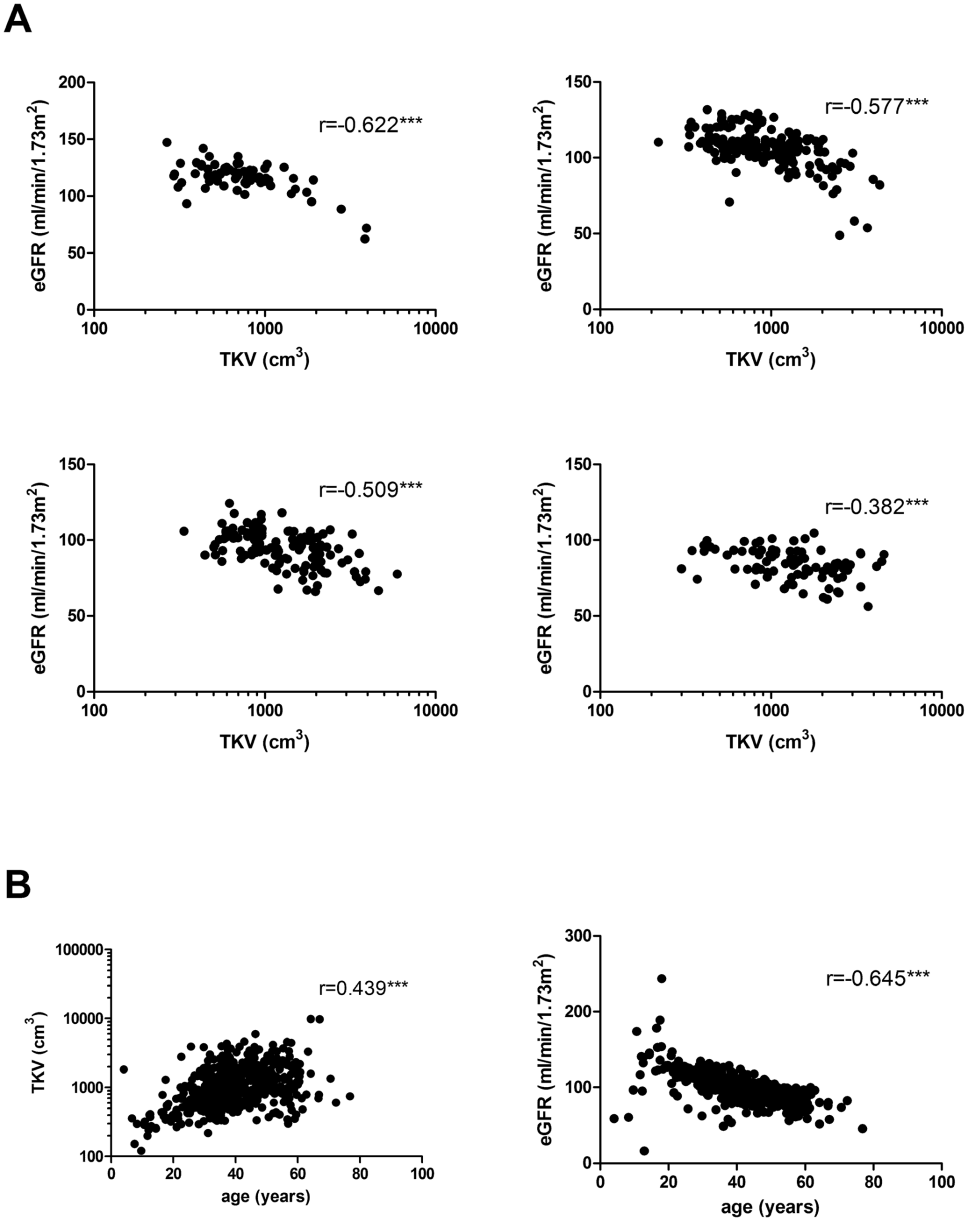
Correlations among TKV, eGFR and age in ADPKD patients. A) Scatter plots between baseline TKV and eGFR. The upper left panel shows patients of age 19–30 years (n = 72), the upper right panel patients of age 31–40 years (n = 171), the lower left panel patients of age 41–50 years (n = 127), and the lower right panel patients of age 51–60 years (n = 90). B) Scatter plots between age and baseline TKV (n = 532) (left panel), and between age and eGFR (n = 502) (right panel).

**Figure 6:**
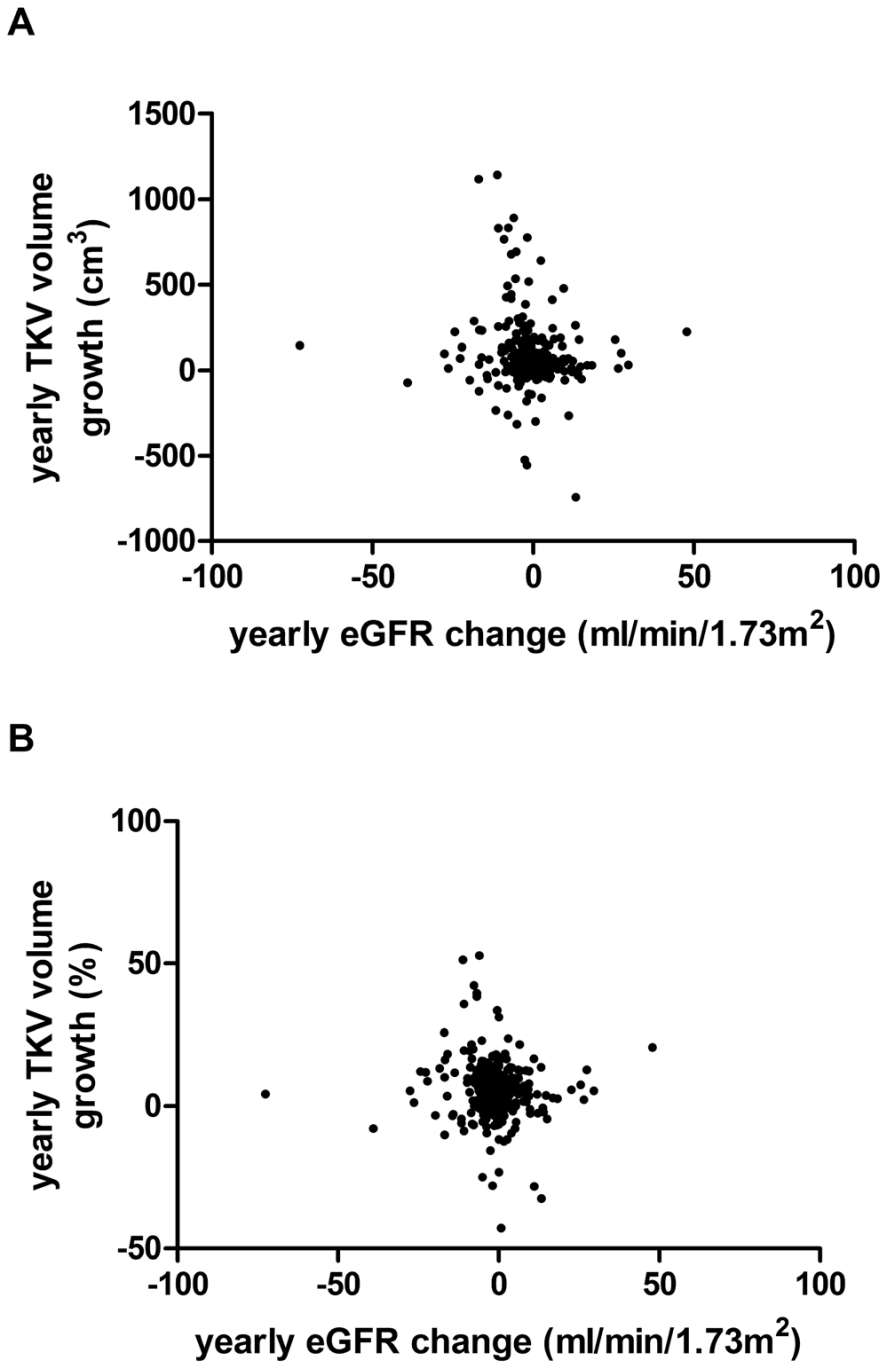
Correlations between yearly TKV growth and eGFR change in ADPKD patients. A) Scatter plot between yearly eGFR change and yearly TKV volume growth (cm^3^) (n = 372). Spearman’s rho is –0.119 (*P* = 0.022). Only patients >18 years and ≤60 years are included. B) Scatter plot between yearly eGFR change and yearly TKV volume growth (%) (n = 372). Spearman’s rho is –0.055 (*P* = 0.288). Only patients >18 years and ≤60 years are included.

There was a significant negative correlation between baseline TKV and eGFR for the different age categories between age 19 and 60 (Fig. 5A). For all patients between age 19 and 60 the *r* amounted to –0.596 (p<0.001). TKV correlated best with eGFR in the age group 19–30 (*r* = –0.622) and was least in the age group 51–60 (*r* = –0.382). As mentioned above, age correlated positively with log_10_-transformed TKV (*r* = 0.439) and negatively with eGFR (*r* = –0.645) (Fig. 5B). However, there was only a very weak correlation between the yearly eGFR change and the yearly TKV volume growth (Fig. 6A and 6B).

### Analysis of predictors of progression

Table 4 shows the results of the linear regression with yearly eGFR change as the dependent variable. The model explains only 19% of the total variance. We identified a highly significant association of log_10_ protein/creatinine ratio and also baseline log_10_ TKV with the yearly decrease of eGFR. Age and thrombocyte count were also significantly associated. At first sight the influence of history of hypertension and intake of antihypertensive drugs on eGFR change seems complicated. When developing the model we found that the association between the unadjusted estimate of history of hypertension and the yearly eGFR change was negative. When controlling for intake of antihypertensive drugs the association turned into a positive one. Since the intake of antihypertensive drugs may reflect a higher disease severity, the addition of this latter parameter to the model seems to be justified.

**Table 4:** Linear regression model with 97% windsorized yearly eGFR change as dependent variable.

Coefficients	Estimate	Standard error	*P*-value	Change of coefficient by	Adjustment in yearly eGFR change (ml/min/1.73m^2^/year)
Log_10_ protein/ creatinine	–4.572024	0.941120	<0.001	1%	–0.020
Log_10_ baseline TKV	–4.992699	1.478459	<0.001	1%	–0.022
Thrombocyte count	–0.012648	0.005818	0.031	1 G/l	–0.013
Age	–0.113218	0.046569	0.016	1 year	–0.113
History of hypertension	2.350887	1.223246	0.056	Presence vs. absence	2.350
Intake of antihypertensive drugs	–1.970294	1.199285	0.102	Presence vs. absence	–1.970
Baseline eGFR	–0.036897	0.034818	0.290	1 ml/min/1.73 m^2^	–0.037
Intercept	20.274693	7.778006	0.010		

Adjusted R^2^ was 0.1943, F-statistic was 10.58 on 7 and 271 DF (*P*<0.001).

Table 5 shows the results of the linear regression with percental yearly TKV change as dependent variable. This model explains only 16% of the variance. Interestingly, age was identified as a highly significant predictor for reduction in the percentage rate of TKV, possibly as a reflection of a higher probability of cyst ruptures. In addition, the intake of antihypertensive drugs, male sex, lower thrombocyte count and log_10_ protein/creatinine were significant predictors for yearly TKV change. Although the dependent variable already accounted for baseline TKV insofar it represents the percental increase, baseline TKV was also retained in the regression model. Paradoxically, a lower diastolic blood pressure was associated with a higher yearly TKV change. It is however difficult to estimate the true effect of blood pressure on TKV change since in most hypertensive patients, blood pressure is controlled by antihypertensive drugs. The regression model for TKV change was hampered by heterogeneity of variance, which could be reduced considerably by introducing observation time into the model.

**Table 5:** Linear regression model with 96.4% windsorized yearly TKV change as dependent variable.

Coefficients	Estimate	Standard error	*P*-value	Change of coefficient by	Adjustment in yearly TKV change (%/year)
Log_10_ protein/creatinine	2.552477	1.295340	0.050	1%	0.011
Log_10_ baseline TKV	3.315135	1.961758	0.092	1%	0.014
Thrombocyte count	–0.014728	0.007203	0.042	–1 G/l	0.015
Male sex	1.846426	0.806099	0.023	male vs. female	1. 846
Intake of anti-hypertensive drugs	2.157377	0.884859	0.015	presence vs. absence	2.157
Age	–0.235288	0.065427	<0.001	1 year	–0.235
Diastolic blood pressure	–0.083163	0.040440	0.041	1 mm Hg	–0.083
Baseline eGFR	–0.072392	0.053032	0.173	1 ml/min/1.73 m^2^	–0.072
Observation time	1.634095	0.905810	0.072	1 year	1.634
Intercept	18.597347	11.763321	0.115		

Adjusted R^2^ was 0.1612, F-statistic was 6.935 on 9 and 269 DF (*P*<0.001).

## Discussion

A majority of patients with ADPKD reach end-stage renal disease (ESRD) in the 5^th^ or 6^th^ decade of life [14], [15], but renal insufficiency may occur much earlier or later. While years ago it was felt that the course of polycystic kidney disease was totally unpredictable [16], more recent studies have shown that mutations in *PKD1*, male sex, early onset hypertension and macrohematuria, baseline GFR, albuminuria, renal cyst volume and uric acid are among the most important factors which predict more severe disease progression and an earlier onset of ESRD [8]–[11], [17]–[23].

Large cross-sectional and retrospective studies of ADPKD cohorts have provided fundamental information on the clinical course and the predictive factors, but their value has been limited by the lack of prospective data for GFR decline and cyst growth. Thus it is only recently – and particularly in association with the advent of CT- and MRI-based radiological techniques that allow precise measurement of renal volume changes – that truly prospective cohorts have been set-up to elucidate the characteristics of polycystic kidney disease progression. The invaluable CRISP cohort has been established between 2001 and 2005 and represents the most detailed cohort of 241 patients with ADPKD [8], [24]. Recently, 6- and 8-year follow-up data have been reported, providing important insight regarding the factors which determine the decline in GFR and TKV growth [10], [25].

Here we describe a large longitudinal clinical cohort of 541 Chinese patients with ADPKD. Our data allow not only verification but also extension of the data from CRISP [8]-[10] and other cohorts [11], [12], [26]. The Chinese cohort is as detailed as the CRISP cohort, yet more than twice the size and specific for the Chinese population, but with only a short observation time. Contrasting with CRISP [8] and the smaller SUISSE ADPKD cohort [11], the Chinese cohort spans a broader age range which includes pediatric and older patients, with a majority of patients (n = 497) in a wider adult age range (19–60 years). This has the advantage to provide prospective data for additional age categories, but introduces some heterogeneity of the data, wherefore we have limited the analysis of the progression data to the age range between 19 and 60. Limitations of the cohort are its short follow-up time, the reliance on estimated rather than measured GFR, the lack of mutational analyses, and the fact that the study population is confined to China.

In agreement with the CRISP and the SUISSE ADPKD results, our data confirm that ADPKD is more severe in male than in female patients, and that there is an age-dependent decrease of eGFR and increase in TKV which extends to older patients. Consistent with the two other cohorts we found similar values for eGFR and TKV and the yearly changes thereof in their respective age range. Thus at a mean age of 32.4±8.9 years, CRISP reported a baseline TKV of 1076±670 cm^3^ and a 5.3±3.9% yearly increase of TKV [8]. At a mean age of 31.2±6.4 years, SUISSE ADPKD reported a TKV of 1003±568 cm^3^ and an extrapolated yearly TKV growth of 5.4±9.5% [11]. Although not precisely comparable we also found a mean TKV around 1000 cm^3^ and an average yearly TKV growth of approximately 5.2% in the age category between 19 and 40 years. Furthermore the baseline eGFR and the yearly eGFR changes seen in CRISP and SUISSE ADPKD were similar to the Chinese patients in the respective age categories. Taken together this suggests that the impact of the Chinese ethnicity on ADPKD is negligible. As a consequence, clinical trials in Chinese ADPKD patients might rely on similar assumptions regarding the definition of clinical endpoints.

Earlier prospective studies in patients with ADPKD have shown that GFR and kidney volume correlate [8], [10], [26]–[28]. Although the follow-up time in our cohort was short (14.6±10.6 months in the adult population of 19–60 years) we could compute the yearly changes in TKV and eGFR in an extended number of age categories. An important finding was that many patients displayed a pronounced and unpredictable creatinine rise and eGFR decline, and this was apparent in all age categories. This suggests that it remains difficult to predict the course of the renal function in individual patients. Furthermore, steep increases in TKV were also found in all age categories and especially in patients with baseline TKV greater than 1500 cm^3^. For reasons not yet well understood it appears that the predictability of the change in TKV is as difficult as the predictability of changes in eGFR. This is also illustrated by the poor correlation between the yearly eGFR and TKV changes in our cohort, and by the data in Figure S2 which show that there are large excursions (increases and decreases) of TKV growth which do not correlate well with increasing baseline TKV. As longer follow-up times become available in our cohort the sudden volume changes - which could be due to hemorrhage or cyst rupture – might become less obvious, and the average TKV growth rate should become more reliable.

The linear regression analysis revealed that the yearly decrease of eGFR was significantly associated with higher log_10_ protein/creatinine ratio, log_10_ baseline TKV and age. On the other hand the linear regression analysis with percental yearly TKV change as dependent variable revealed that the intake of antihypertensive drugs, male sex, lower thrombocyte count and higher log_10_ protein/creatinine were associated with this variable. The fact that the TKV change was dependent on observation time implicates that the variance is larger for patients with short observation periods and decreases with longer observation periods. This might possibly be explained by the fact that bursting cysts lead to lower TKV at shorter observation periods but less so when the observation time is longer. With increasing observation time, the trend towards a steady TKV growth might get clearer. Consequentially and as stated above, it is essential for the study of TKV growth to maximize follow-up times in order to reduce the “noise” caused by ruptured cysts.

An important finding which has not been described in other cohorts was the identification of a reduced thrombocyte count in older age groups. Although there is a small decrease of the thrombocyte count with increasing age in the normal population, we found a 32% lower thrombocyte count (–71 G/l) in the oldest age group when compared with the youngest cohort patients. Furthermore we found a positive correlation between thrombocyte count and eGFR, and a negative correlation with TKV. Bath et al. also described a reduced number of thrombocytes in ADPKD patients (average age 31 years) in comparison with matched control subjects (–63 G/l), in addition to an increased platelet volume (median 8.4 vs 8.0 fl), suggesting that there is enhanced platelet consumption in ADPKD [29]. Our regression analysis showed that the TKV growth rate was significantly influenced by the thrombocyte count, suggesting that platelets might be implicated in the pathogenesis of cyst growth. Further studies need to be performed to define the pathogenic role of thrombocytes in ADPKD.

In conclusion, we describe the clinical characteristics and the factors that predict disease progression in a large cohort of Chinese patients with ADPKD. Among the progression factors we found that log_10_-transformed TKV and protein/creatinine ratio significantly predicted eGFR loss and were associated with TKV growth. Furthermore we identified the decreased thrombocyte count as a novel parameter which is associated with more advanced renal impairment, higher TKV and higher TKV growth. The Chinese cohort provides an important data source for the understanding of ADPKD disease progression and the design of future clinical trials in China.

## Supporting Information

Figure S1 **Frequency distributions for different categories.** A) frequency distributions for age (years) (n = 541); B) frequency distributions for number of prescribed antihypertensive drugs (n = 522); C) frequency distributions for diastolic blood pressure (DBP) in mm Hg (n = 403); D) frequency distributions for systolic blood pressure (SBP) in mm Hg (n = 403); E) frequency distributions for estimated glomerular filtration rate (eGFR) in ml/min/1.73 m2 (n = 502); F) frequency distributions for total kidney volume (TKV) <5000 in cm3 (n = 529).
(TIF)

Figure S2 **Correlations between baseline TKV and yearly TKV growth.** A) Baseline TKV (cm^3^) lined up in ascending order, with corresponding yearly TKV growth (cm^3^) depicted in the graph above. B) Yearly TKV growth (cm^3^) lined up in ascending order with corresponding baseline TKV (cm^3^) depicted in the graph above. Only patients >18 years and ≤60 years are included (n = 393).
(TIF)

File S1 **Supporting Tables.** Table S1: Table shows the eGFR and the yearly changes of the eGFR, stratified by age categories. The eGFR was calculated according to the CKD-EPI formula in adults and according to the Schwarz formula in children. Table S2: Data show mean ± standard deviation. Table S3: Table reports baseline kidney volumes in cm3. RKV, right kidney volume; LKV, left kidney volume; TKV, total kidney volume. Table S4: Table reports baseline cyst volumes in cm3. RCV, right cyst volume; LCV, left cyst volume; TCV, total cyst volume.
(DOC)
