# Supplementary material for: Clinical Characteristics and Disease Predictors of a Large Chinese Cohort of Patients with Autosomal Dominant Polycystic Kidney Disease
Source: PLoS One. 2014 Mar 20;9(3):e92232. doi: 10.1371/journal.pone.0092232 (PMC3961326; doi:10.1371/journal.pone.0092232)
Supplement: File S1 — Supporting Tables. Table S1: Table shows the eGFR and the yearly changes of the eGFR, stratified by age categories. The eGFR was calculated according to the CKD-EPI formula in adults and according to the Schwarz formula in children. Table S2: Data show mean ± standard deviation. Table S3: Table reports baseline kidney volumes in cm3. RKV, right kidney volume; LKV, left kidney volume; TKV, total kidney volume. Table S4: Table reports baseline cyst volumes in cm3. RCV, right cyst volume; LCV, left cyst volume; TCV, total cyst volume. (DOC) [file pone.0092232.s003.doc]

**Supporting information**

Table S1

Estimated GFR and changes in eGFR, stratified by age categories, and for the entire cohort

| **Age categories (years)** | ≤18 | 19-30 | 31-40 | 41-50 | 51-60 | >60 | All |
| --- | --- | --- | --- | --- | --- | --- | --- |
| **eGFR (ml/min/1.73 m2)** | | | | | | | |
| N | 20 | 74 | 171 | 128 | 90 | 19 | 502 |
| Mean±SD | 130.9±50.2 | 116.4±13.0 | 105.9±13.1 | 94.2±11.8 | 84.2±10.1 | 75.7±14.6 | 100.4±20.1 |
| Median | 138 | 118 | 108 | 95 | 84 | 79 | 101 |
| IQR | 52 | 12 | 12 | 17 | 13 | 20 | 24 |
| Range | 16-244 | 62-147 | 49-132 | 66-124 | 56-105 | 46-100 | 16-244 |
| **Yearly eGFR changes (ml/min/1.73 m2 per year)** | | | | | | | |
| N | 14 | 58 | 151 | 89 | 75 | 13 | 400 |
| Mean±SD | 7.0±26.8 | 1.3±8.0 | -0.7±7.9 | -1.8±7.1 | -3.5±10.6 | -1.3±2.2 | -0.9±9.6 |
| Median | -2.4 | 0 | -1 | -1.4 | -2.5 | -1 | -1.3 |
| IQR | 31.9 | 5.1 | 6.2 | 5.8 | 4 | 2.8 | 5.5 |
| Range | -91.1 | -59.5 | -66.4 | -54 | -98.1 | -7.9 | -138.5 |

Table shows the eGFR and the yearly changes of the eGFR, stratified by age categories. The eGFR was calculated according to the CKD-EPI formula in adults and according to the Schwarz formula in children.

Table S2

Selected baseline laboratory data of Chinese ADPKD cohort, stratified by age categories, and for the entire cohort

| **Parameter** | Units | N | Age ≤18 | Age 19-30 | Age 31-40 | Age 41-50 | Age 51-60 | Age >60 | All ages |
| --- | --- | --- | --- | --- | --- | --- | --- | --- | --- |
| Sodium | mmol/l | 296 | 140.5±2.7 | 142.0±2.6 | 141.9±2.9 | 142.0±3.0 | 142.5±2.7 | 141.9±3.6 | 142.0±2.9 |
| Potassium | mmol/l | 295 | 4.1±0.2 | 4.2±0.4 | 4.2±0.5 | 4.2±0.4 | 4.3±0.5 | 4.3±0.5 | 4.2±0.4 |
| Calcium | mmol/l | 261 | 2.45±0.23 | 2.42±0.20 | 2.45±0.19 | 2.40±0.17 | 2.42±0.19 | 2.48±0.17 | 2.43±0.19 |
| Phosphate | mmol/l | 244 | 1.53±0.42 | 1.19±0.19 | 1.19±0.25 | 1.16±0.19 | 1.19±0.22 | 1.26±0.23 | 1.20±0.24 |
| Glucose | mmol/l | 355 | 4.6±0.5 | 4.8±0.6 | 5.0±0.6 | 5.1±0.7 | 5.6±1.4 | 5.3±0.8 | 5.1±0.9 |
| Total cholesterol | mmol/l | 369 | 4.0±0.8 | 4.2±0.8 | 4.4±0.9 | 4.6±0.9 | 4.4±0.9 | 4.2±0.8 | 4.4±0.9 |
| LDL cholesterol | mmol/l | 311 | 2.1±0.5 | 2.4±0.7 | 2.4±0.8 | 2.5±0.8 | 2.4±0.8 | 2.3±0.5 | 2.4±0.8 |
| HDL cholesterol | mmol/l | 343 | 1.2±0.3 | 1.4±0.6 | 1.4±0.7 | 1.4±0.5 | 1.5±0.7 | 1.1±0.3 | 1.4±0.6 |
| Triglycerides | mmol/l | 370 | 1.2±0.6 | 1.2±0.7 | 1.4±1.0 | 1.7±1.2 | 1.3±0.7 | 1.5±0.5 | 1.4±1.0 |
| Albumin | g/l | 415 | 49.6±6.9 | 48.7±3.8 | 47.2±3.8 | 46.4±3.8 | 45.6±3.5 | 46.3±2.9 | 46.9±3.9 |
| Hemoglobin | g/l | 401 | 123.5±27.9 | 144.9±18.6 | 135.7±17.7 | 134.0±19.6 | 133.5±17.5 | 131.5±18.1 | 135.5±19.1 |
| Leukocytes | G/l | 400 | 6.0±1.8 | 6.2±1.4 | 6.0±1.7 | 6.1±1.4 | 5.8±1.7 | 5.5±1.1 | 6.0±1.6 |
| Thrombocytes | G/l | 399 | 224.5±73.1 | 225.0±54.2 | 195.2±46.6 | 197.6±54.0 | 183.2±53.0 | 153.4±57.6 | 197.2±54.1 |

Data show mean ± standard deviation.

Table S3

Reference data for baseline kidney volumes, stratified by age categories, and for the entire cohort

| Age (years) |  | ≤18 | 19-30 | 31-40 | 41-50 | 51-60 | >60 | All |
| --- | --- | --- | --- | --- | --- | --- | --- | --- |
| N |  | 23 | 81 | 180 | 134 | 95 | 19 | 532 |
| RKV | Mean±SD | 211±173 | 403±334 | 540±375 | 715±449 | 748±457 | 1077±1302 | 605±489 |
|  | Median (IQR) | 174 (100) | 326 (230) | 427 (386) | 575 (544) | 650 (597) | 612 (661) | 463 (494) |
|  | Range | 52-868 | 122-2073 | 106-2285 | 145-3115 | 140-2276 | 254-4852 | 52-4852 |
| LKV | Mean±SD | 247±218 | 439±330 | 587±388 | 781±490 | 828±518 | 1103±1487 | 660±533 |
|  | Median (IQR) | 177 (94) | 363 (214) | 468 (428) | 620 (579) | 694 (731) | 574 (628) | 495 (555) |
|  | Range | 70-966 | 143-1859 | 113-2495 | 150-2849 | 153-2433 | 202-5430 | 70-5430 |
| TKV | Mean±SD | 458±385 | 842±646 | 1126±739 | 1495±909 | 1575±948 | 2180±2774 | 1265±1002 |
|  | Median (IQR) | 359 (184) | 693 (444) | 863 (790) | 1234(1112) | 1361(1324) | 1349 (924) | 954(1008) |
|  | Range | 121-1835 | 267-3932 | 219-4324 | 336-5964 | 300-4594 | 482-9839 | 121-9839 |

Table reports baseline kidney volumes in cm^3^. RKV, right kidney volume; LKV, left kidney volume; TKV, total kidney volume.

Table S4

Reference data for baseline cyst volumes, stratified by age categories, and for the entire cohort

| **Age (years)** |  | ≤18 | 19-30 | 31-40 | 41-50 | 51-60 | >60 | All |
| --- | --- | --- | --- | --- | --- | --- | --- | --- |
| **N** |  | 23 | 81 | 180 | 134 | 95 | 19 | 532 |
| **RCV** | Mean±SD | 79±164 | 222±319 | 341±333 | 507±430 | 552±413 | 880±1245 | 410±456 |
|  | Median (IQR) | 29 (54) | 125 (215) | 228 (347) | 393 (456) | 442 (523) | 422 (515) | 276 (452) |
|  | Range | 0-777 | 6-1957 | 5-1818 | 0-2936 | 12-1953 | 106-4439 | 0-4439 |
| **LCV** | Mean±SD | 108±200 | 242±294 | 378±341 | 565±465 | 637±472 | 931±1388 | 459±494 |
|  | Median (IQR) | 39 (51) | 190 (181) | 276 (398) | 454 (500) | 527 (593) | 481 (655) | 307 (504) |
|  | Range | 3-889 | 13-1779 | 9-1998 | 5-2386 | 14-2314 | 22-5126 | 3-5126 |
| **TCV** | Mean±SD | 187±359 | 464±602 | 719±652 | 1072±857 | 1189±858 | 1811±2613 | 869±929 |
|  | Median (IQR) | 78 (107) | 320 (389) | 483 (707) | 840 (921) | 991 (1087) | 1142 (944) | 605 (963) |
|  | Range | 8-1667 | 28-3737 | 14-3319 | 49-5322 | 38-4267 | 147-9295 | 8-9295 |

Table reports baseline cyst volumes in cm^3^. RCV, right cyst volume; LCV, left cyst volume; TCV, total cyst volume.
